# Care obligations, employment and mental health

**DOI:** 10.1093/eurpub/ckac130.187

**Published:** 2022-10-25

**Authors:** U Rose, H Burr

**Affiliations:** Work & Health, Federal Institute for Occupational Safety and Health, Berlin, Germany

## Abstract

**Background:**

The presentation is dedicated to employees who care for others in addition to their work. The starting point is the representative cohort study on mental health at work (S-MGA), which covered both care at home and care outside the home in the second wave of the survey. In this regard, cross sectional associations with exhaustion and work-life balance as indicators of mental health were examined taking into account full-time and part-time employment.

**Methods:**

The sampling frame consisted of all German employees being subject to social security contributions and born between 1951 and 1980. The baseline sample consisted of 4511 survey participants of whom 1279 males and 1358 females were asked for informal care at the follow-up interview. Employment conditions as well as work-life balance were obtained by personal interview; exhaustion was obtained in a paper and pencil questionnaire. Statistical analysis was conducted descriptively and in linear and logistic regressions stratified by gender and controlled for age.

**Results:**

Informal care at home was reported by n = 74 individuals (2.8%) and care outside of their home by n = 236 (8.9%). The association between both types of care with exhaustion was below the level for significance. There was an increase of work-life-imbalance for females caring at home but not for those who were caring outside. For males there were no effects of both types of care. Including the part-time/full-time distinction indicator within the regression models showed that women who cared at home had lower exhaustion scores and lower work-life imbalance when they were employed part-time.

**Conclusions:**

The results show that caring at home for females leads to work-life imbalance and that part-time employment mitigates the negative effects on work-life imbalance and exhaustion. However, there are strong limitations by the sample size and the number of observations at the second wave of assessment.

**Key messages:**

- The results show that caring at home among females seems to lead to work-life imbalance.
- Part-time employment seems to mitigate the negative effects on work-life imbalance and exhaustion.

